# A Rare Case of a Renal Cell Carcinoma Confined to the Isthmus of a Horseshoe Kidney

**DOI:** 10.1155/2015/126409

**Published:** 2015-06-22

**Authors:** Michael Kongnyuy, Daniel Martinez, Anthony Park, Barrett McCormick, Justin Parker, Mary Hall

**Affiliations:** ^1^University of South Florida Morsani College of Medicine, Tampa, FL 33612, USA; ^2^James A. Haley Veterans' Hospital, Tampa, FL 33612, USA

## Abstract

Horseshoe kidney (HSK) is the most common renal anomaly. Reports of the incidence of renal cell carcinoma (RCC) in HSK are conflicting. Very few cases of isthmus-located RCC have been reported in the literature. We report a unique case of an isthmus-located RCC. Proper vascular and tumor imaging prior to surgery is key to successful tumor removal.

## 1. Introduction

Horseshoe kidney (HSK) occurs in 3% of the population and is the most common renal fusion anomaly [1–4]. There are conflicting reports as to whether or not there is an increase incidence of renal tumors in HSK compared to the normal population [2, 4, 5]. There however have been very few reported cases of RCC located at the isthmus of an HSK per our literature search. We report a rare case of renal cell carcinoma (RCC) located at the isthmus of an HSK as well as an aberrant vascular supply to the isthmus and tumor.

## 2. Case Report

**Figure 1:**
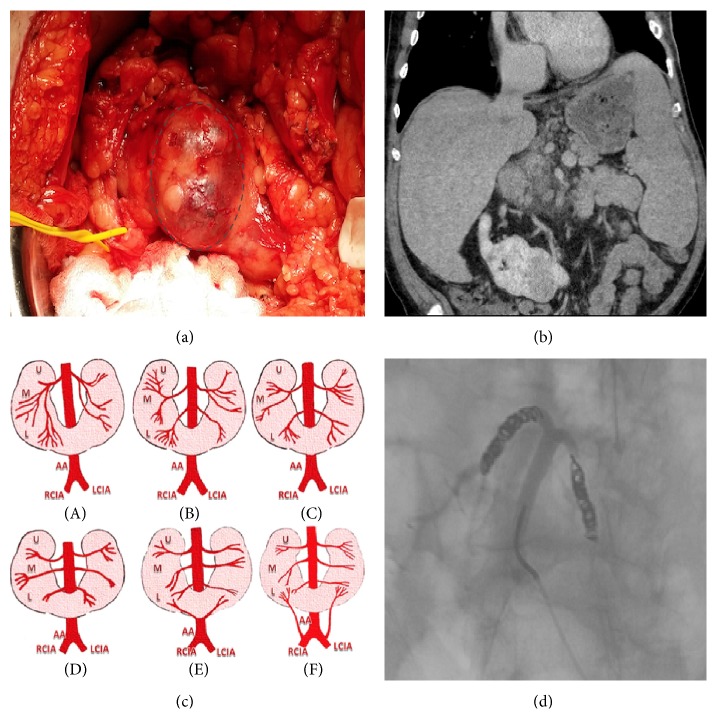
(a) Encircled intraoperative gross specimen of resected RCC containing isthmus. (b) Coronal CT image indicating RCC containing isthmus. (c) Catton of six major patterns of HSK vasculature. (d) Aberrant single infrarenal vascular supply of the isthmus of HSK with coils at the bifurcation.

A 73-year-old man presented for presurgical evaluation of a biopsy proven 4.2 cm pT3aNxMx clear cell renal cell carcinoma (RCC) mass at the isthmus of his horseshoe kidney. Prior to surgery, renal ultrasound was performed that showed a minimally hypovascular space-occupying mass at the junction of the inferior pole of right renal component of the HSK and renal isthmus which corresponded to a prior CT angiogram of his abdomen (Figure 1(b)). Renal arteriography delineated the arterial supply of the HSK. We recommended open bilateral partial nephrectomies resection of the isthmus and tumor.

Arteriography prior to surgery was indicated to further evaluate renal and tumor vasculature. Two left and right renal arteries were noted to supply the two kidneys and a midline artery arising from the infrarenal aorta bifurcated to supply the isthmus/tumor (Figure 1(d)). Given the solid arterial supply to the tumor, proactive measures to minimize intraoperative bleeding were taken. Interventional radiology embolized the midline artery supplying the isthmus and tumor.

A flexible cystoscopy was performed to rule out any possibility of bladder cancer that could also contribute to the patient's hematuria. Pollack catheters were placed bilaterally for intraoperative identification of the ureters. The coils used to embolize the midline arterial branches, supplying the isthmus, were palpable, facilitating arterial clipping proximal to the coils. The partial nephrectomies were then performed, with an estimated blood loss of 600 cc.

The resected tumor and isthmus weighed 78 g and measured 6.4 × 5.3 × 4.1 cm with a tan to brown, focally friable, nodular, and well-encapsulated 4.3 × 3.7 × 3.3 cm mass (Figure 1(a)). Pathology showed narrow but negative margins and a Fuhrman Nuclear Grade 3 clear cell RCC with papillary features.

## 3. Discussion

Horseshoe kidney is the most common renal fusion anomaly and more common in males than in females [2, 5]. HSKs are usually asymptomatic and are often incidentally discovered during physical exam or seen on imaging studies; when symptomatic, they are associated with hydronephrosis, infection, or calculus formation [2, 6]. When located at the isthmus, these can mimic intra-abdominal disease processes like aortic abdominal aneurysm or ovarian tumors [2, 7, 8].

It is not clear if the incidence of RCC in HSK patients is increased compared to non-HSK individuals, as there are conflicting reports in the literature [2, 4, 5]. Our patient presented with RCC of the isthmus of an HSK, with less than ten reported cases, per our literature search using search words like HSK tumors, isthmus tumors, and HSK complications.

Most HSK tumors are corrected with bilateral partial nephrectomies via division of the isthmus [9–11]. Our patient underwent a similar procedure but in this case it was not a simple division of the isthmus but a total excision of a tumor-containing isthmus. Surgical removal of a tumor in the isthmus can be technically challenging [9] with an increased risk of intraoperative bleeding due to aberrant vasculature [6, 11]. The blood supply to most HSKs has been classified into 6 different patterns [3]. The isthmus often is supplied by a dual arterial supply with each half of the isthmus having a dedicated arterial vasculature (Figure 1(c)) [6]. Our patient's vasculature did not fall into any of the 6 patterns. There was a single infrarenal arterial branch supplying both the tumor and the 2 halves of the isthmus (Figure 1(d)).

Our patient's surgery required a thorough preoperative preparation. Several imaging modalities (arteriogram, CT angiogram, and ultrasound) were performed, which showed a hypovascular tumor in the isthmus (Figures 1(b) and 1(d)). Hypovascularity is likely due to a single arterial blood supply, as opposed to the typical 2 or more arterial branches that would supply the isthmus. Preoperatively, interventional radiology embolized the 2 branches of the midline artery. Intraoperatively, vascular surgery was present to ensure that the coils were in place and that there were no further vascular concerns (Figure 1(d)).

## 4. Conclusion

Horseshoe kidneys are rare but are the most common renal fusion anomaly. They present a surgical challenge due to their varied vascular supply. An example was this patient's vascular aberrancy at the isthmus. RCC of the isthmus is also a unique finding that has rarely been reported in the literature. The surgical treatment of renal lesions in the horseshoe kidney continues to be a challenge due to the variety of pathology and vascular anomalies that can occur.
